# Mobilities I: Crisis

**DOI:** 10.1177/03091325251352538

**Published:** 2025-06-30

**Authors:** Cristina Temenos

**Affiliations:** 5292 University of Manchester, UK

**Keywords:** Climate, crisis, economic geography, geopolitics, health, mobilities

## Abstract

In this report on mobilities, I discuss research published between 2017-present, focussing on how multiple crises intersect with mobilities. I examine how recent work in health, economic, ecological, and geopolitical geographies are shaped by crisis mobilities. Focussing on the relationship between crisis moments and on-going crisis states, I examine how recent work on crisis mobilities drives mobilities research forward while also working towards spatialising understandings of crisis. I suggest that mobility is not just about physical movement but also about navigating the temporalities of ongoing hardship, where crisis is part of the lived experience rather than an exceptional event.

## I Crisis mobilities

Since the last Progress Reports on mobilities (Merriman, 2015, 2016, 2017), interdisciplinary work on movement, speed, friction, and flow has proliferated. Unfolding events have precipitated this: the global COVID-19 pandemic; a cost of living crisis fuelled by uncertain and exponential inflation rates; increasingly evident effects of ecological and climate breakdown; and geopolitical upheavals that include a global rising right-wing populism as exemplified by the election wins of Trump, Meli, Modi, and Meloni among others; genocides in Dafur and Palestine; the Russian invasion of Ukraine as well as more than 110 armed conflicts globally.^1^ In short, the world is experiencing multiple, increasing, and intersecting crises, which have profound and wide-ranging effects on mobilities across all scales and regions. In this report, I demonstrate how crisis moments often force a reconsideration of mobility systems, such as transportation networks, migration, and access to essential services. Crisis disrupts as well as reconfigures mobility by challenging existing infrastructures and prompting new forms of movement. The report demonstrates that crisis, however, is not only exceptional but must also be considered through a dyadic relationship, attending to the mundane and ‘ordinary’ conditions of ongoing crisis. I suggest that mobility is not just about physical movement but also about navigating the temporalities of ongoing hardship, where crisis is part of a lived experience rather than an exceptional event. Highlighting the relationship between crisis as event and as ongoing state demonstrates how mobility is shaped by everyday precarity, contributing to events and processes and conditions for polycrisis.

Crisis, as a term in geographical research, is widely used though theorisations often remain siloed. Crisis geographies have focused largely on economy (Dixon, 2023; Fraser, 2014; Harvey, 1989; Leitner, 1989; Piven, 1983), disaster (Adey et al., 2015; Cretney, 2017; Donovan et al., 2024), and more recently everyday crises (Barker, 2020; Dimitrakou and Ren, 2025; Temenos, 2022). Crisis can be split any number of ways. Sheller (2018), for example, highlights three crises as inherently linked to mobility/immobility: the urbanisation crisis, the migration crisis, and the climate crisis. She argues that these crises are intertwined and dependent on the unequal power dynamics that govern (im)mobilities. Meyer (2020) contends that crises are generally understood as falling within three distinct areas: economic, ecological, and social. For both Meyer and Sheller, urbanisation is linked to climate breakdown through the production of cities, suburbs, and hinterlands, as well as the social and racialised segregation that occurs, not only in urban space, but showing that it is more widely reproduced in land use across urban-rural territories. Social, economic, and ecological crises are intertwined then, via the ways that they play out spatially.

Temporally, crisis is largely thought of in two ways, as a disruptive moment, a flashpoint that shifts fundamental understandings or processes, or as a prolonged and ongoing condition (Roitman, 2013). A moment of crisis can simultaneously speed up actions, cause unintended consequences, and immobilise people and political, social, or economic processes. Meanwhile ongoing crisis conditions, what Berlant (2011) called the crisis-ordinary, often have the inverse effect, slowing down larger processes of mobility and creating a sense of waiting or an impasse, a state of recalibration and suspension, for those experiencing it. At the same time, on-going conditions of crisis can create a focus on the micro-mobilities of individual bodily movement (Harris et al., 2019). The seemingly inverse relationship of sudden crisis events and long-term crisis conditions highlights the importance of speed as an integral relation to analyses of global mobility patterns, of people, pathogens, things, and ideas (Marvin et al., 2023). Mobilities scholars have undertaken these analyses through the lens of assemblage (Baker and McGuirk, 2017; McCann and Ward, 2012), constellations (Cresswell, 2010), and conjuncture (Davidson and Ward, 2024; Hart, 2024; Peck, 2024; Thompson et al., 2025). In a state of intersecting crises, exploring the relationship between the crisis moment and crisis-ordinary is crucial for understanding how contemporary mobilities are shaped (Temenos, 2022). The remainder of this report examines intersections of crisis mobilities across health, economic, ecological, and geopolitical landscapes.

## II Health

In March 2020, the world seemingly stopped. People were ordered to shelter at home in isolation. Political movements such as the Estallido Social in Chile, the country-wide protests at growing socio-economic inequalities in the country, were indefinitely interrupted (Woods, 2022). The first global lockdown saw a record decline in carbon emissions (Fiske et al., 2024; Nguyen et al., 2025). The rise of mutual aid groups saw a renewed focus on the importance of local networks (Cresswell, 2021; Ho and Maddrell, 2021). And the role of localised forms of active transport, particularly cycling, became a focus of hope (Mostafanezhad, 2020; Nachman et al., 2023). As SARS-CoV-2 emerged and spread throughout the world from autumn 2019, to the global lockdowns of March 2020, the rapidity of this ‘viral mobility’ was paraleled first by a slowing down and then stopping of many other forms of mobility, from personal mobilities during the lockdown, logistical mobilities of global shipping, to automobilities both personal and logistical (Adey et al., 2021: p 1; Lin, 2022; Nikolaeva et al., 2023). Imposed immobility fuelled a collective discussion about how this crisis could be an opportunity to enact permanent structural and lifestyle changes to enhance the health of people and the environment (Gibson, 2021; Mostafanezhad, 2020). The COVID-19 pandemic as a health crisis could engender forms of experimentation to create lasting progressive or even radical transitions (Hoolihan et al., 2022; McGuirk et al., 2021; Temenos, 2022; Travlou, 2021; Verhulst, 2023).

And yet, from the beginning of the first lockdowns, it was clear that (im)mobilities were experienced unequally across multiple axes of class, race, gender, and geographical locations (Adey et al., 2021; Borkowski et al., 2021; Cresswell, 2021; Glodenau et al., 2021; Manzo and Millnelo, 2020; Rose-Redwood et al., 2020; Vecchio and Tiznado-Aitken, 2020). Those experiencing high levels of socio-economic deprivation, including relatively low-paid service and delivery workers, were often compelled to commute and interact with others at a time when the global population was told to stay at home and isolate from SARS-CoV-2 (Arias-Molinare et al., 2023; Glodenau et al., 2021). Sparke and Williams (2022) dubbed COVID-19 a ‘Neoliberal Disease’. Physical mobility of individuals during the pandemic came down to a question of survival and social reproduction. Those with no economic savings, precarious housing, or no option for remote work were subject to higher infection rates. The result was an increase in socio-economic inequalities caused by the COVID-19 lockdowns at a planetary scale. As Shaw and Waterstone (2021: 1788) note ‘the richest saw their wealth increase during the COVID-19 pandemic, for the first time in 25 years, extreme global poverty rose, with around 45% of global humanity surviving on less than $5.50 a day’ (see also World Bank, 2020: 39).

The COVID-19 Pandemic is not the only health crisis affecting mobility, and mobility in various forms can greatly affect the spread of disease. Ali and Keil (2011) demonstrate how globally networked infrastructures of cities contributed directly to the spread of SARS in 2003. Globalisation to expand the reach and increase the speed of the movement of capital also exploded localised disease ecologies, rapidly propelling them across the world before local public health teams could identify, let alone put in measures, to contain quick spreading airborne diseases. Health crises generate the movement of people but also of ideas. For example, the HIV crisis in the 1980s and 1990s, together with the opioid overdose crisis in cities in North America during the 1990s, led to the adoption and expansion of needle exchanges and other harm reduction programs globally (Brown, 1999; McCann and Duffin, 2023; McCann and Temenos, 2015). Attempts to immobilise disease resulted in mobilising ideas, policies, practices and models to stem the spread of HIV. HIV itself was and remains a disease inextricably linked to migration and mobility across borders (Cassels et al., 2023; Di Feliciantonio, 2020). Iaquinto (2020: 177) notes diseases operate as ‘viral travel companions’ to people as they move. Viral mobilities can in turn be understood through geographies of marginalisation. They are linked to migration, and the increased spread of viruses having disproportionately negative effects on those with lower socioeconomic status. This includes people who are unable to move to access better forms of healthcare, or conversely those who lose access to care due to forced migration (Ehrkamp, 2017).

## III Economic crisis and mobility

Economic crisis is one of the richer veins in which the concept of crisis has been taken up in geography. The dominance of political economic approaches to understanding contemporary spatial practices is predicated on Marxist analyses of capitalist crises of overproduction. Tidal forces pushing the overproduction of commodities create surpluses which devalue the end product, causing producers to search for new forms of investment. The conditions of crisis occur because much of the value is already embedded in the means of production, fixed in particular landscapes, and therefore unable to be liberated in search of new technology or profit (Harvey, 2018[1982]). Ebbing profit margins drive unemployment and push down wages, causing crises of social reproduction, yet freeing up capital for new investment, beginning the cycle anew. Harvey (2010: p 90, italics added) reminds us that ‘Capital, Marx insists, is a *process of circulation* and not a thing’.

Capital then is inherently mobile. Its attendant crises embedded into this process are in and of themselves forces of mobility. The ebb and flow of capitalist economic crises are mirrored by how crises of social reproduction play out, interspersing immediate crisis moments, such as the Great Recession, within the longer moments of the crisis ordinary (Jupp, 2022; Marvin et al., 2023; Temenos, 2022, 2024). These cyclical crises often entail an imperative of the crisis moment, when something fundamental shifts, and the ways that it plays out for people and societies experiencing hardship deepens and expands ongoing inequality (Brickell, 2020; Harris et al., 2019; May et al., 2020).

Economic crisis spurs mobility across scales. Housing crises and transportation patterns illustrate these processes. These range from extreme crises of increased homelessness (Dozer, 2020; Rady and Sotomayor, 2024) to wider financial hardship that changes housing decisions. For example, from whether university students might be living at home and thus engaging in longer commuting times, choices to attend school based on proximity to the family home, and the ways that people’s mobility is constrained or imposed on them vis-à-vis their housing and employment options (Cadima et al., 2020; Censi, 2021; Ortega-Alcazar and Wilkinson, 2021; Ravalet et al., 2017; Salazar, 2021). The wider financialization of housing further limits who has access to (mobile) capital, credit, and debt to be able to buy or rent in housing markets globally (Fernandez and Aalbers, 2020; Fields, 2017; Sokol and Pataccini, 2020). Financialization thereby presents a paradox for mobilities scholars. Financialization allows capital to flow more freely and therefore enables greater mobility. It overcomes Harvey’s spatial fix to the extent that fixed assets, such as homes, are understood to have primarily financial rather than socially reproductive uses. And yet, financialization has been shown to foreclose such mobility to a larger proportion of the population, concentrating wealth and constraining both financial as well as physical movement.

Housing and transport changes during and after economic crisis are not limited to the most recent global financial crisis. There is a long history of economic crisis spurring both voluntary and involuntary mobility at both the wealthy and impoverished ends of societies. Dozer (2020: 755) writing on how homeless encampments emerged from the conjuncture of the financial crisis of the 1970s and ongoing structural racism that kept Black communities impoverished states: ‘The spatial contours of these moments of crisis are structured into carceral strategies such as poor camps and hospitalisation, vagrancy laws and imprisonment, social service hubs or skid rows, and shelter warehousing’. In this analysis, strategies of enclosure and carcerality engaged in the interplay between the involuntary immobility of Black people, often unable to find housing outside of specific neighbourhoods, as well as the forced dispossession of housing that came with economic crisis and uncertainty. Crisis pushed people into encampments, simultaneously holding them in a state of mobility through the impermanence of the camp and the immobility of carceral capitalist logics enacted through crisis.

## IV Ecological mobilities

It is not just housing or economic crises that spur migration, whether at the macro or micro scale. The climate is changing, with increasingly unpredictable and exponential shifts in extreme weather events, ocean currents, land surface temperatures, habitat loss, soil erosion, and mass extinction. Climate migration equally is not only about climate refugees as depicted in either policy talk (McNamara and Gibson, 2009; Ransan-Cooper et al., 2015) or pop culture (Gergan et al., 2020; Whyte, 2018). Climate mobility is shaped by political processes of bordering (Boas et al., 2024). Borders in and of themselves are not fixed, but mutable, particularly in instances of climate breakdown and emergency. Borders are a relation in and of themselves, containing different meanings dependent on who and in what context they are encountered (Bambilla and Jones, 2020). Migration in the face of climate crisis has been put forward as one of the major forms of adaptation, yet questions have been raised about how feasible and to what end such forms of climate mobility can or should be depended on, and indeed how those most affected by climate change might resist the assumption of their future mobility as necessary adaptation (McNamara and Gibson, 2009; Simpson et al., 2024).

The multiple facets of the climate and other intersecting crises incite geographers to engage in equally multiple and intersecting methodologies to grapple with it. Sultana (2021) demonstrates the conjunctural nature of the climate crisis, with that of disaster capitalism (Klein, 2007), and the COVID-19 pandemic (Nissen and Cretany, 2024). Within social movements, whether related to climate or health, a hierarchy of crisis often emerges (Nissen and Cretany, 2024). Here the interlinkages of multiple intersecting crises and their attendant mobilities need to take on a more pluralistic engagement of geography. Addressing multiple crises encompasses several methodological strategies including taking a more spatio-historic lens, a focus on the relationship between macro and micro processes, thinking through how planetary processes affect local places, and how to encourage interdisciplinarity. In doing so, geographers can overcome the all too frequent qualitative-quantitative divide. Moreover, geographers must take seriously post and decolonial methodologies and alternative world views to engage a more wholistic and generative analysis of crisis mobilities and responses (Sheppard, 2022).

Climate mobilities not only encompass that of the spectacular events that cause movement but also consider mobilities of the everyday. Introducing the concept of the ‘ordinary Anthropocene’, Fredriksen (2021) recounts the loss and return of Florida’s wild flamingos. Returning not to a pristine nature reserve, or even a reclaimed and restored wetland, these birds chose as their habitat Palm Beach County’s Stormwater Treatment Area 2, a human-made wetland built for the express purpose of filtering pollutants. Fredriksen (2021) defines the ordinary Anthropocene by the ‘ongoing, everyday more-than-human relationships, actions and less-than-planetary assemblages through which the Anthropocene is sensed and lived’ (Fredriksen, 2021: 532). She goes on to chart how the very idea of the Anthropocene itself causes momentum to push foraward certain ideas and practices of crisis (Fredriksen, 2025). Like Berlant’s use of the term ‘crisis ordinary’, the ordinary Anthropocene attaches a slower, more quotidian temporality to ways of understanding ongoing ecological uncertainty and crisis. It offers an analytic to think through more-than-human mobilities in a multi-relational constellation of ongoing and intersecting crises, both the sudden and immediate, as well as the slower, everyday movement in times of ecological uncertainty.

The conjuncture of crises, particularly socio-ecological crises at the intersection of the crisis-ordinary and the spectacular crisis-moment, has also been understood through the framing of precarity (Petrova, 2024). Such framings foreground the political processes that have brought about crisis states and crisis moments, and indeed, precarity in this sense is understood as an ongoing, politically induced position that keeps environments in a constant state of suspended animation. Forced climate migration due to rising sea levels, desertification, or rampant wildfires is not only experienced by human communities but also by more-than-human inhabitants of these places.

## V Geopolitical mobilities

Crises, whether geopolitical, environmental, health, social, or economic, have long served as flashpoints that force a reckoning with the systems that structure everyday mobility. As Merriman et al. (2017) note, military mobilities have historically been underexamined in the political geographies of mobility; yet in the years since, scholars have turned attention to the complex entanglements of military, civilian, and logistical movements, particularly during times of heightened instability. Political geographers have considered mobile military technologies (Jackman and Brickell, 2022), embodied and more than human military mobilities (Bormpoudakis and Bourlessas, 2022), and the politics inherent between the relationship of military and civilian mobilities in times of both war and peace (Veal, 2020). In their historical account of the Greek Civil War, Bormpoudakis and Bourlessas (2022) demonstrate how the examination of displacement in mobile military geographies highlights the multiple human-environment relationships that are moulded by both territory and terrain, evoking the importance of both mobility and immobility during times of geopolitical crisis.

Territory, as a primary focus of geopolitics, in and of itself, is not immobile. Borders move all the time. Sometimes it is the very territory, the land or river itself that shifts through both terrestrial and anthropogenic events (Thomas, 2017). And at other times, borders are moved by people redrawing them, diplomatic agreements, and military and colonial occupation (Diener and Hagen, 2022). These border movements are not merely symbolic; they materially reconfigure access to land, to services, and to life itself. The shifting borders of Palestinian and Ukrainian territory are notable in contemporary geopolitics for bearing witness to centuries old political conflicts and contestations that remain unresolved, contributing not only to border movements, but to movements of people and goods at global scales. This accounts for logistical systems, the movement of food, medicine, and other essential items into crisis ridden and occupied land. Using countermapping as method, Abushama (2024) charts the ways in which settler-colonial practices converge with global capital to encroach and occupy Palestinian land, shifting and shrinking its borders in the process. We witness how settler colonialism and imperial aggression produce layered crises of mobility. These infrastructures do not merely enable movement; they also delimit it, enclosing populations while facilitating the circulation of capital and military force. The building of modern roads and highways act as technologies of occupation and simultaneously establish borders, demonstrating how Palestinian ‘geographies remain prone to rupture and transformation’ (Abushama, 2024: np). Interdependencies are laid bare by geopolitical crises. They crystalise how mobility systems are not only sites of technical coordination but sites too of political struggle. Moments of crisis then, render visible the infrastructures that underpin mobility, calling into question whose movements are secured and whose are suspended. It is in these moments that the politics of care, governance, and resistance are most sharply articulated, demanding a rethinking of how mobility is governed and to what end.

## VI Conclusion

Any progress report on crisis mobilities cannot claim to be comprehensive. Progress reports could be written individually for each of these four areas. And of course, the report could have been organized around various axes of crisis and mobility: climate migration, emergency, catastrophe, and vital and viral mobility, among other topics (Adey, 2024a; Ali et al., 2022; Baldwin et al., 2019; Gold, 2020; Iaquinto, 2019; Sodero, 2019; Sodero and Sinclair, 2024). What emerges from this progress report is a view to understanding the multiple instances and intersections of mobilities in times of polycrisis. The notion of crisis is complex, and it involves the relationships between crisis as a moment that changes things instantly, and the ongoing state of living in precarity, or the crisis ordinary. Mobilities in times of crisis are equally complex. Mobilities involve transnational as well as local, sometimes even hyper-local landscapes in and through which mobility is enabled and constrained. It is undertaken voluntarily or through forced movement. Specific constellations of mobility are always affected through governance and politics. Looking forward, it is essential for scholars to think through new ways of governing mobility in times of crisis and uncertainty. Collectivity, mutual aid, and commoning have offered interesting and important ways to think through governance and its challenges in the polycrisis (Adey et al., 2023; Mould et al., 2022; Nikolaeva et al., 2019; Sheller, 2018).

Examining the ways in which mobility is encountered, for example, through the lens of emergency (Adey, 2024b), risk (Gibson and Warren, 2024), radicality (Davidson, 2021), justice (Sheller, 2018; Verlinghieri and Schwanen, 2020), and transformation (Adey et al., 2023) are important as we face the current conjuncture. Methods of studying mobilities research in geography and adjacent fields are another area of importance to help us think through the import and material and theoretical stakes with which contemporary mobilities studies grapple under the current intersecting crises.

Mobilities are relational to both everyday and eventful crises which are constantly affecting the present moment. As I’ve shown, contemporary geographical understanding of mobility is mediated through a state of constant insecurity. Ongoing precariousness affects mobilities on both individual and infrastructural scales, and in so doing, shapes crisis responses in as much as crisis responses shape mobilities. The emergence of the polycrisis, where multiple and overlapping crises tend to amplify each other, did not happen suddenly. Rather as Berlant (2011) notes, crises accumulate over time. They accumulate through micro movements of politics and policies, big and little migrations, and the slower ebb and flow of everyday life.

Each crisis, whether economic, social, political, or environmental, does not occur in a vacuum. Rather, they compound. In turn, these intersecting crises affect social reproduction, political and economic systems, and environmental conditions, constructing the current conjunctural moment, and (re)shape public life as well as private lives. They can spur new forms of politics, as Halvorsen and Angelcos (2025) have recently noted. Mobilities in the context of polycrisis, then, are not only reactions or formations of individual events, but shape contemporary geographies of crisis, politics, and the everyday. Thus, geographical scholarship and methodological approaches to studying mobilities must be attuned to the relationship between eventful and everyday crises.

## Note

1. At the time of writing, the Geneva Academy estimated more than 110 armed conflicts globally. https://geneva-academy.ch/galleries/today-s-armed-conflicts.
